# Upcycling of Commercial Biobased Polyester Bioplastics—Turning Used or Spoiled Materials into Added-Value Products

**DOI:** 10.1021/acspolymersau.6c00048

**Published:** 2026-04-27

**Authors:** Vojtěch Jašek, Silvestr Figalla, Radek Přikryl

**Affiliations:** Institute of Materials Chemistry, Faculty of Chemistry, 48274 Brno University of Technology, 61200 Brno, Czech Republic

**Keywords:** upcycling, bioplastics, polyesters, PHA, PLA, PBS, biobased materials, valorizing, depolymerization

## Abstract

Bioplastics incorporate different renewable sources via direct biosynthesis or biotechnological intermediates, contributing to the eventual production of a biobased system. Sustainable polymers and materials represent an essential substitute for petrochemical intermediates and the synthesis of applied compounds. We listed, described, and discussed three main groups of commercially applied and produced bioplastics: polyhydroxyalkanoates (PHAs), polylactic acid/polylactide (PLA), and polybutylene succinate (PBS). Although all the mentioned biobased polymers possess biodegradability at specific conditions, their waste or spoiled primary products have significant potential for new and diverse applications. The thermally degraded, hydrolyzed, or physically aged biopolyesters lack sufficient performance in their initial applications; upcycling is an alternative and promising strategy that produces valorized, added-value products or complex multistep-derived functional systems. This comprehensive review describes the major challenges that the discussed biobased polymers face, identifies fundamental chemical approaches for bioplastics as entering substances, and outlines the potential and outlook for the waste-based molecules and precursors produced. The eventual alternative applications include the pharmaceutical industry, the cosmetics segment, the production of chemical intermediates and reactants for organic synthesis, and various functional and circular materials, such as recyclable thermoplastics and thermoset vitrimers.

## Graphical abstract

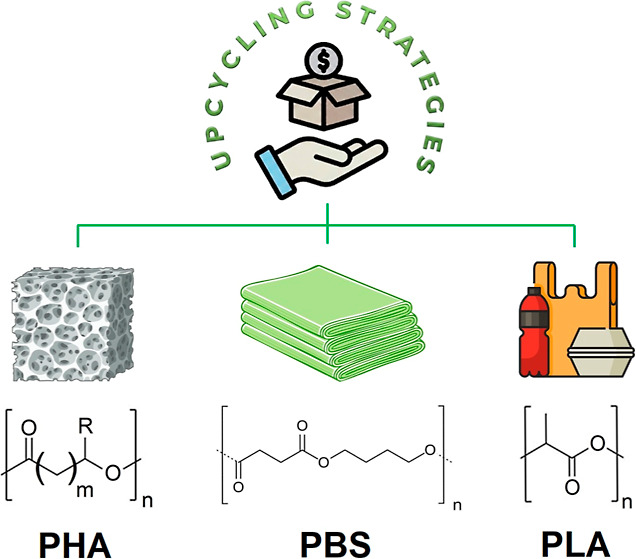

## 1 Introduction

Biobased polymers and their products in the materials industries represent key alternatives to various fossil-based substances across countless utility and application fields. Their role and purpose are linked to numerous production-related and environmentally affecting factors. The evolving legislation plays a crucial role, particularly for the European Union and its partners, as the EU strategy moves toward industrial manufacturing and incorporates waste from recycled sources into final products.^1–3^ Moreover, the overall reduced human-generated carbon footprint is also taken into account in the plastics and materials industry (limiting CO_2_ emissions).^4,5^ Strategies, holding enormous potential to fulfill these expectations, are often connected to the biotechnology industrial sector, which produces most of the entering substances or even the complete final products applied in particular fields.^6^ In terms of polyesters, the biotechnological production of alcohols/polyols and carboxylic acids is essential for the potential bioplastics industry.^7,8^ The total bioplastic manufacturing reached 2.36 million tons in 2021, of which 1.55 million tons represented degradable materials (primarily polylactide (PLA), polyhydroxyalkanoates (PHA), and thermoplastic starch (TPS)) and 0.85 million tons were nondegradable materials and plastics (biobased polyethylene (Bio-PE) and biobased polyethylene terephthalate (Bio-PET)).^9,10^ The projected global production capacities of bioplastic forecasting values in 2028 reach 7.4 million tons in total, from which 4.6 million tons should be degradable bioplastics (197% increase) and 2.8 million tons represent nondegradable bioplastics (229% increase).^11^

Polyhydroxyalkanoates (PHAs) are a large group of biopolyesters produced in vivo by microbial producers as a carbon source.^12^ From the bioplastics standpoint, this polymer group is connected exclusively to the biotechnological sector (involving the downstream—the isolation and purification approach leading to the pure polymer). It does not include any postproduction chemical synthesis or modification approach, unlike other polyester compounds.^12–14^ PHAs are produced via various bacterial producers, such as *Cupriavidus necator* (formerly *Ralstonia eutropha*),^15^ *Pseudomonas* species (*P. putida* and *P. oleovorans*),^16,17^ or *Bacillus* species (*B. megaterium* and *B. subtilis*).^18,19^ The microbiological producers vary based on the accumulated PHA’s carbon backbone chain length.^15–19^ Since PHA is a large, multicomponent group of compounds, the carbon backbone chain length and functional group location vary across PHA members.^20^ Figure 1 contains a general structural formula of PHAs, marking the carboxylic backbone by parameter “*m*”. In contrast, the potential terminal functional group represents the parameter “*R*”. Generally, the ester functional group located in the C3 carbon (*m* reaches value one and *R* represents methylene −CH3) exhibits rigid character due to the high crystallinity, while monomers with longer carboxylic chains and ester bonding located further from the carboxylic functional group (*m* reaches values 2–4 and *R* represents hydrogen) possess more flexible and elastic character. Poly­(3-hydroxybutyrate) (PHB or P­(3HB)) is the biggest and most well-known group of PHA, containing a C4-long carbon backbone and the esterified hydroxyl group bound to the C3 carbon.^21^ Many other frequently used and investigated PHAs are copolymers of individual monomers. 3-Hydroxyvaleric acid (3-HV) is a common copolymerized compound containing a C5-long carbon backbone and a hydroxyl functional group located on the C3 carbon. Poly­(3-hydroxybutyrate-*co*-3-hydroxyvalerate) (PHBV) is a widely used copolymer of 3-hydroxybutyric acid and 3-hydroxyvaleric acid, overcoming P3HB’s rigidity and crystallinity.^22^ Therefore, PHBV represents a more flexible and rigid alternative to the homopolymeric P3HB.^23^ 4-Hydroxybutyric acid (4-HB) is another different hydroxyl group-containing carboxylic acid possessing a C4-long carbon backbone. Unlike P3HB, the functional hydroxyl group is bound to the C4 carbon; therefore, the commonly used and produced copolymer poly­(3-hydroxybutyrate-*co*-4-hydroxybutyrate) (P­(3HB-*co*-4HB)) contains two different monomeric structures with varying compositions, resulting in much lower crystallinity and enhanced flexibility.^24^ As for 2023, PHAs find their application potential in five major fields: packaging (≈40%), biomedical sector (≈25%), cosmetics (≈20%), agriculture (≈10%), and other applications (≈5%).^25^ The production costs are significant challenges for PHAs. In particular, P3HB is often mentioned as a substitute for polypropylene (PP),^26^ and their production costs differ significantly—PP costs around $1.2-$2.5/kg, whereas P3HB costs around $3.5-$4.5/kg.^27^ Due to this economic factor, PHAs have market potential in high-value-added applications, such as the biomedical sector and cosmetics.^28,29^ Biocompatibility ensures unique utility potential for bone tissue engineering,^30^ while biodegradability represents a promising environmental solution for microplastics in cosmetics (e.g., for exfoliation, texture, or product stability).^31^

**Figure 1:**
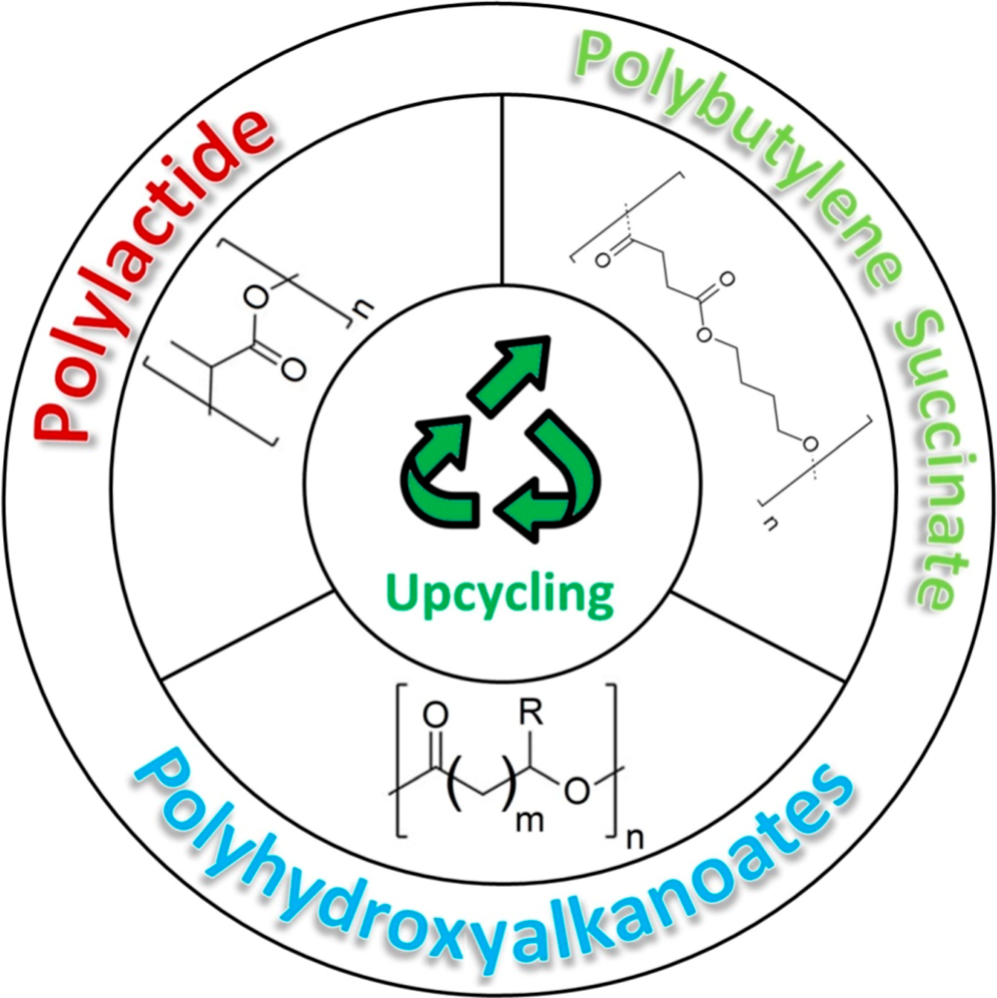
Three types of commercially produced and applied polymers represent the bioplastic segment.

Polylactic acid (or polylactide) (PLA) represents a homopolyester containing lactic acid (2-hydroxypropanoic acid) as a monomer unit.^32^ The term “polylactide” is often used in the literature due to the synthetic approach applied for PLA production.^33–35^ Lactic acid, as a biotechnological product, serves as an entry compound for PLA production, and several feedstock types are used for its production (corn starch, sugarcane, or various food wastes).^36–38^ Although the direct polycondensation of lactic acid was investigated and verified, the process poses many challenges that complicate scalability and profitability (especially the high process temperatures of around 200 °C and the considerable vacuum levels required to ensure water removal).^39,40^ The synthesis of lactide, followed by ring-opening polymerization (ROP), is a more efficient process in the current PLA industry.^39,40^ Lactide manifests a cyclic lactic acid dimer formed from two lactic acid molecules via a nucleophilic substitution mechanism called intramolecular transesterification (backbiting)—the PLA oligomer is formed during the process via condensation, and the acid end group attacks the ester linkage, forming lactide, which is isolated via distillation.^41–43^ The formed lactide is eventually homogenized with a suitable catalyst (mainly tin­(II) 2-ethylhexanoate (Sn­(Oct)_2_) and used for PLA’s synthesis through ROP (that is why the term “polylactide” often describes the eventual polymer).^43^ Packaging (food and beverage) accounts for the majority of the total PLA market (≈55–60%),^44^ followed by other application segments, such as textile/nonwoven, 3D printing, medical, electronics, and agriculture.^45–47^ The crystallinity of PLA mainly determines its mechanical properties, which are important for different utilities. Since lactic acid forms two stereoisomers, L-(+)-lactic acid and D-(−)-lactic acid, the crystallinity and overall performance are highly dependent on PLA’s composition.^48,49^ While pure monostereoisomer PLA exhibits high crystallinity (up to 50%), the racemic PLA is mainly amorphous (crystallinity below 10%).^50^

Unlike PHAs, derived completely by microorganisms,^12^ and PLA (synthesized from single biotechnologically produced lactic acid),^32^ polybutylene succinate (PBS) is manufactured from two biotechnologically obtainable compounds—1,4-butanediol (BDO) and succinic acid (SA).^51^ BDO is produced from several renewable biomass-based entering substances (carbohydrates and agricultural waste)^52,53^ using particular microbiological producers: *Klebsiella, Bacillus*, or genetically modified *Saccharomyces cerevisiae*.^54–56^ BDO’s biotechnological production exemplifies an alternative to the fossil-based butanediol synthesized via the acetylene route (which involves a two-step process—acetylene reaction with two formaldehyde molecules producing 2-butyne-1,4-diol, followed by the hydrogenation)^57^ or the maleic anhydride route (butane is oxidized to form maleic anhydride, which is esterified and hydrogenated to form 1,4-butanediol).^58^ Succinic acid (SA) is biotechnologically produced from different entering biomass types (sugars, glycerol, or the biomass waste)^59,60^ by the particular microorganisms (genetically engineered *Escherichia coli* or *S. cerevisiae*).^61,62^ While the upstream (fermentation) generally comprises identical steps to other productions mentioned earlier, the downstream differs due to the phase-solid form of SA.^63^ Unlike the liquid lactic acid solutions or BDO, SA is separated and purified via crystallization.^64^ The petrochemical route to SA involves butane oxidation to maleic anhydride followed by hydrogenation.^65^ The condensation process to PBS involves specific esterification catalysts, such as titanium-based titanium­(IV) butoxide or tin-based tin­(II) 2-ethylhexanoate.^66^ The esterification is performed at high temperatures (170–250 °C) and high vacuum levels (1–10 torr).^67–69^ Unlike both short-chain PHAs (mainly P3HB) and PLA, PBS exhibits strong potential for flexible materials due to the low glass transition temperature (ranging from −40 °C to −10 °C)^70,71^ and exceptional elongation at break (hundreds of percent).^72^ The leading PBS application fields are packaging (flexible films),^73^ agriculture (mulching films),^74^ automotive (potential lightweighting),^75^ and medical (implants or drug delivery).^76^

This proposed work focuses specifically on upcycling products using bioderived macromolecular systems. In addition to upcycling, reusing, energy recovery, and other strategies for processing polymeric waste and nonperforming original products, closed-loop chemical recycling exemplifies an essential, prospective, and attractive way to circulate the polymers originally applied.^173–175^ Circular chemical recycling involves depolymerizing the originally used macromolecular systems, producing products chemically identical to the starting materials, typically with a high molecular weight similar to that of the virgin polymer. The advantage of such a process is the closed-loop circularity of materials streams, which does not complicate the transformation into different application fields.^173–175^ However, chemical recycling typically involves multiple processing steps, including depolymerization, purification, preprocessing, and eventual polymerization. This multistep, complex processing requires considerable engineering and costs. Upcycling approaches may exemplify simpler, less expensive pathways to the final products, making them an attractive strategy for polymeric valorization.^176^ Since this work focuses on three polymeric ester materials, PHAs, PLA, and PBS, the closed-loop chemical recycling was successfully performed with PLA and PBS, especially due to their sufficient thermal stability.^159,177^ The depolymerization step requires a suitable reactive system, such as water (hydrolysis), alcohol (alcoholysis), or an amine (aminolysis), which undergoes nucleophilic substitution, resulting in the formation of the specific monomer.^178,179^ The monomer(s) are processed and circularly polymerized into the original macromolecule. PHAs exhibit poor thermal stability at typical working temperatures (especially above 120 °C), undergoing spontaneous β-elimination that produces unsaturated carboxylic acids, making them unsuitable for circular polymerization.^180,181^ Therefore, PHAs cannot be circularly repolymerized like PLA or PBS. That is why chemical upcycling may represent a promising strategy for the production of useful added-value products, valorizing waste and spoiled macromolecular bioderived polyesters.

Since all the introduced polymers are biodegradable, primarily via a hydrolysis mechanism catalyzed by specific esterases and depolymerases,^77,78^ their chemical stability in the polymeric state is inferior.^79^ The environmental conditions for the biodegradable process are essential. PHAs are easily degradable under aerobic/anaerobic conditions and at ambient temperatures (20–30 °C).^109^ PLA requires specific conditions: high temperature (60 °C and higher), high humidity, specific microorganisms and enzyme producers, and hydrolyzable conditions.^186,187^ PBS requires high-temperature composting (60 °C and higher) and degrades much slower in marine conditions compared to PHAs.^188^ Instead of the mechanical recycling, leading to the decrease of the mechanical properties and devaluing the materials,^80,81^ the upcycling approach may occur to produce added-value products based on the used waste polyester bioplastics.^82^ Upcycling is generally defined as a process toward new items or products not connected to the original purpose but exhibiting higher quality, value, or function compared to the starting materials.^83^ Since all the introduced polyesters lose most of their applicability due to their structural instability, their chemical structures may serve as templates for new and added-value products.^84^ This work (see Figure 1) focuses on the starting biobased bioplastic identification from their original application standpoint, followed by the summarized investigated and verified upcycling approaches performed in the currently upheld studies.

## 2 Upcycling of Polyhydroxyalkanoates

Polyhydroxyalkanoates represent a large group of different polymers, as was summarized in the Introduction section of this work. Poly­(3-hydroxybutyrate) exemplifies the most applied and well-known representative of this group.^85,86^ Therefore, this section is dedicated primarily to P3HB, since this homopolymer is currently produced on an industrial scale and its applicability and availability have recently been vigorously investigated.^87^ The (in)­stability is P3HB’s unique property, but it complicates its processing and application potential.^88^ P3HB is accumulated in the microorganism as a reserve carbon source, which is used during stress conditions.^89^ The poor thermal stability of specific PHAs, determined by their molecular structure and the thermodynamics of elimination and nucleophilic substitution reactions, complicates the processing required by the plastic industry.^90^ The overall structural instability causes poor thermal resistance, which is a key processing parameter due to the conventionally applied melt techniques, such as extrusion, injection molding, blow molding, or thermoforming.^91–93^ Beta-elimination (β-elimination) is the essential mechanism underlying P3HB’s thermal instability, involving collapse of the polyester structure and the generation of crotonic acid as an undesired secondary product. The mechanism of β-elimination involves a proton removal from the C3 carbon atom (the β-carbon) by the carboxyl functional group, followed by the ester linkage breakdown (the esterified oxygen atom forms a bond with the carbonyl carbon, resulting in the carboxyl functional group formation).^94^ The double bond occurs in the polyester structure, eventually reaching the carbon backbone end, and crotonic acid is formed when the ester end group undergoes β-elimination.^94–96^ Not only does the mechanism lead to P3HB chain shortening and eventual breakdown, but the resulting crotonic acid exhibits a highly unpleasant odor and potential risks.^97^ The β-elimination process is mainly determined by the processing temperature, but the catalysts (acids/bases) and the plasticizers present in the product significantly affect the degradation process.^98,99^ Many published investigations focused on the P3HB processing window, which is a specific condition during thermoplastic processing.^100–102^ The melting point of P3HB is around 175–180 °C, so the optimal processing temperature for minimizing β-elimination is typically 180–190 °C.^23,87^ The process window can be modified by blending different polymers with P3HB, such as poly­(3-hydroxybutyrate-*co*-4-hydroxybutyrate) (P34HB),^103^ or incorporating additional monomers into the structure, such as dilinoleic succinate.^104^

A perspective PHA upcycling approach producing added-value, degradable, and recyclable poly­(ether ester) structures was reported by Li et al. (see Figure 2).^105^ The authors described and synthesized a promising bicyclic monomer, 4-methyloctahydro-2H-benzo­[b]­[1,4]­dioxepin-2-one (4-MOHB), derived from P3HB via efficient steps. Initially, they alcoholyzed P3HB using ethanol as the nucleophile and sulfuric acid as the acidic catalyst. The product, ethyl 3-hydroxybutyrate (HBEt), was obtained at 75%. The same depolymerization approach was applied to the P3HB-*co*-P4HB copolymer; however, multiple catalysts were investigated, including sulfuric acid (66% yield of HBEt), ferric trichloride (39% yield of HBEt), and hydrochloric acid (11% yield of HBEt). Additionally, the hydrochloric-acid-catalyzed process was performed with methanol (representing the nucleophile), yielding 72% of methyl 3-hydroxybutyrate (HBMe). Then, the synthesis of ethyl 3-((2-hydroxycyclohexyl)­oxy)­butanoate followed, involving the produced HBEt, cyclohexene oxide (CHO), and boron trifluoride-ether (BF_3_·Et_2_O). The oxirane-opening reaction produced a 68% yield of ethyl 3-((2-hydroxycyclohexyl)­oxy)­butanoate (3-(2-HCH)­OBEt). The final synthesis of the proposed polymerizable monomer structure, 4-methyloctahydro-2H-benzo­[b]­[1,4]-dioxepin-2-one (4-MOHB), involved a basic-catalyzed hydrolysis of 3-(2-HCH)­OBEt, producing ((2-hydroxycyclohexyl)­oxy)-butanoic acid (3-(2-HCH)­OBA) (yield 85%), followed by the intramolecular esterification of the hydrolyzed product. After esterification, the final monomer product, 4-MOHB, was obtained at 88%. The overall conversion from P3HB to 4-MOHB was 38%. Finally, the polymerization of 4-MOHB was conducted in a high-purity nitrogen atmosphere, catalyzed by tin­(II) 2-ethylhexanoate (Sn­(Oct)_2_). The chemical depolymerization of poly­(3-MOHB) was performed using *p*-toluenesulfonic acid (TsOH) and an elevated temperature (80 °C) to confirm the circularity of the synthesized polymer. The authors achieved a 79% yield of 4-MOHB. All products were structurally verified by ^1^H NMR, ^13^C NMR, FT-IR, and ESI-MS techniques. The authors declare that the produced poly­(4-MOHB) exhibited an amorphous state (*T*_*g*_ between 28 and 35 °C), high thermal stability (TGA onset at 263 °C), and a number-average molecular weight (*M*_n_) of up to 14 kDa, with D̵ around 1.20–1.25.

**Figure 2:**
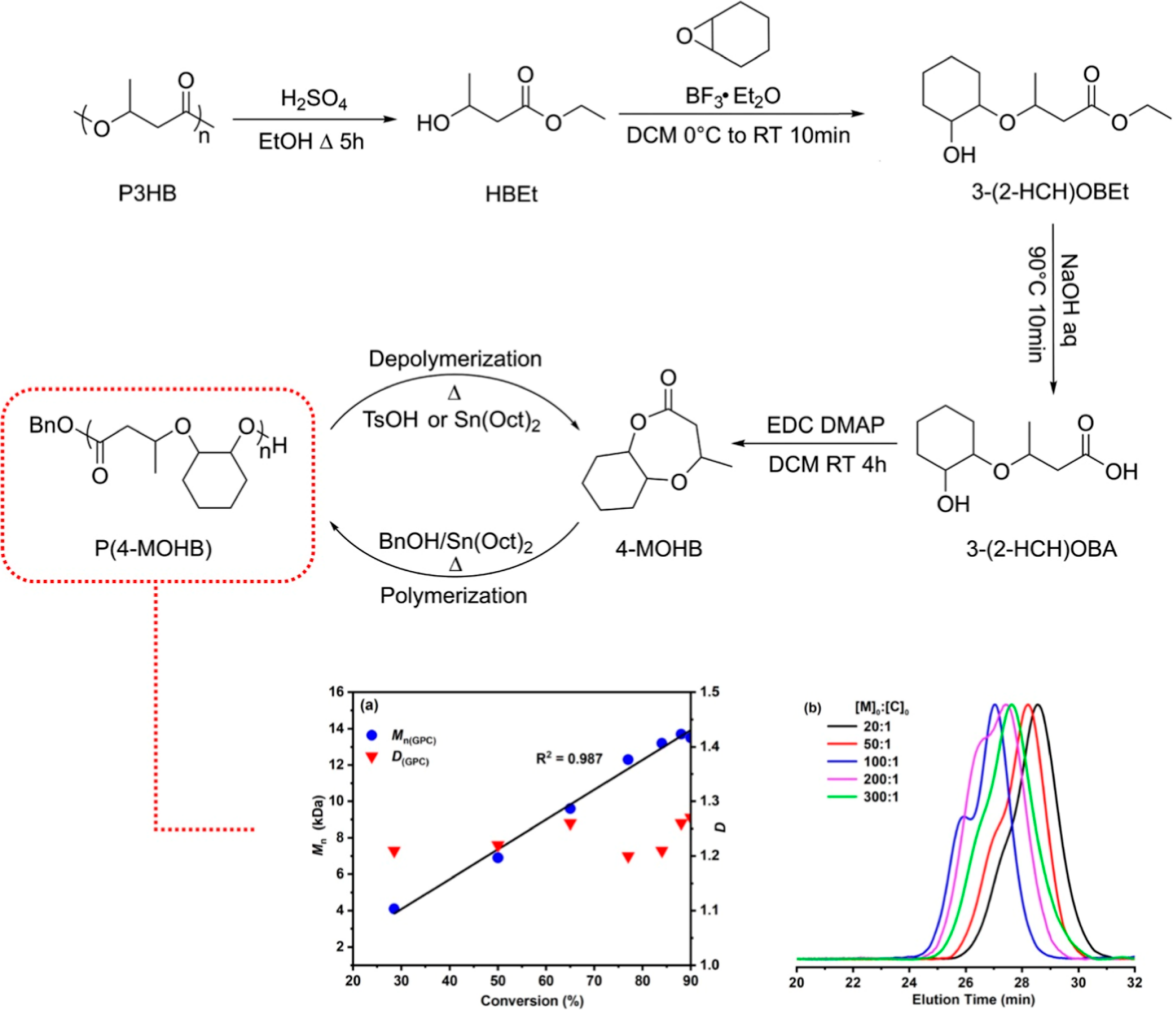
Chemical upcycling of P3HB through a multistep process, yielding a polymerizable monomer, 4-methyloctahydro-2H-benzo­[b]­[1,4]-dioxepin-2-one (4-MOHB), which can be circularly polymerized and depolymerized in a closed-loop approach. (a) The number-average molecular weight/polydispersity dependence on the polymer’s conversion. (b) The GPC graphs determining the length of the synthesized polymers. Reproduced with permission, Copyright 2022, American Chemical Society.^105^

While the previous investigation converted P3HB into monoesters of 3-hydroxybutanoic acid, which served as an entering reactant for a polymerizable P3HB-derived monomer, the following study used the commonly considered adverse process—β-elimination of P3HB—as a prospective upcycling approach (see Figure 3).^106^ The authors performed catalytic pyrolysis of P3HB, producing *trans*-crotonic acid (yield 86%), catalyzed by magnesium oxide. This byproduct underwent amidation with 2-aminoethanol, producing 3-((2-hydroxyethyl)­amino)­butanoic acid (^N^hydroxyethyl-β-homoalanine) (yield 81%). Before the intramolecular cyclization, the secondary amide group, containing one leftover bonded proton, must have been protected, using di*tert*-butyl dicarbonate (Boc_2_O). The synthesized byproduct, Boc-^N^hydroxyethyl-β-homoalanine, obtained via the protective modification, yielded 77%. After the protection, the synthesis of a polymerizable amide-ester monomer, 5-methyl-*N*-Boc-1,4-oxazepane-7-one (MeOxPBoc), followed, involving the protected Boc-^N^hydroxyethyl-β-homoalanine, 1-ethyl-3-(3-(dimethylamino)­propyl)­carbodiimide hydrochloride (EDC·HCl), and 4-dimethylaminopyridine (DMAP). The white crystal polymerizable monomer achieved 80% yield and was polymerized via a ring-opening mechanism (ROP), catalyzed by tin­(II) 2-ethylhexanoate (Sn­(Oct)_2_). Finally, the deprotection of the produced poly­(MeOxPBoc) was performed in dichloromethane (DCM) and trifluoroacetic acid (TFA). In addition to the complete conversion of P3HB into a poly­(amide ester) thermoplastic, the closed-loop depolymerization to MeOxPBoc was verified, involving solution depolymerization catalyzed by Sn­(Oct)_2_. An alternative depolymerization procedure involved a pyrolysis of poly­(MeOxPBoc) catalyzed by zinc chloride at 150 °C and under reduced pressure (2 mmHg). The products were structurally verified using ^1^H NMR, ^13^C NMR, FT-IR, and ESI-MS. Based on the conclusion, the produced poly­(MeOxPBoc) reached varying *T*_*g*_ values between −2.2 °C at 6.8 kDa (M_n_) and 20.6 °C at 69.3 kDa (*M*_n_). The TGA onset temperature reached 234.5 °C, and the inflection point reached 258.4 °C. According to the authors, polymerization exhibited first-order kinetics with an apparent rate constant of *k*_app_ = 0.42 h^–1^.

**Figure 3:**
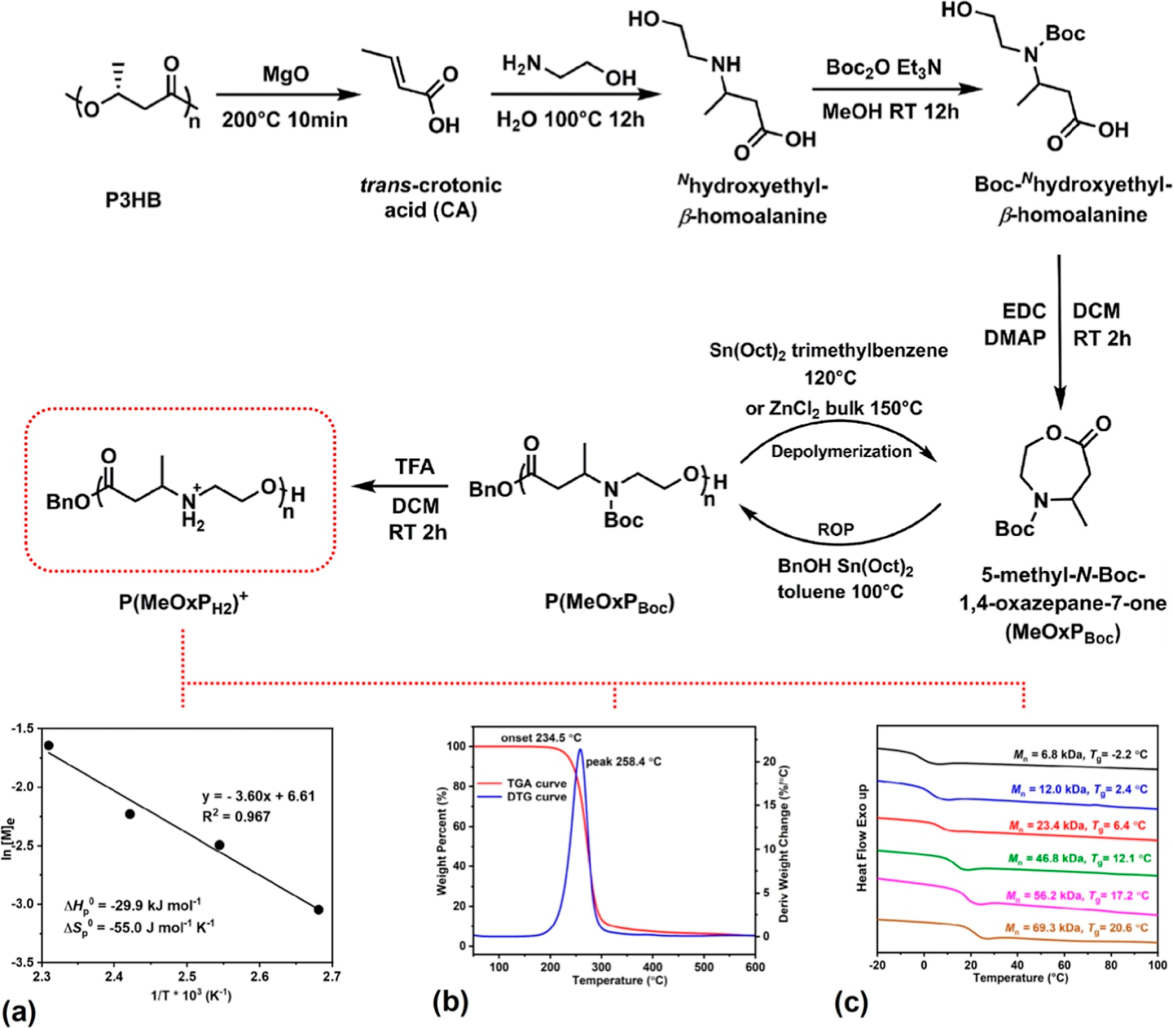
Chemical upcycling of P3HB through the crotonic acid synthesis (CA), followed by its amidation (^N^hydroxyethyl-β-homoalanine), amide protection (Boc-^N^hydroxyethyl-β-homoalanine), intramolecular esterification (MeOxPBoc), amide-ester monomer polymerization (poly­(MeOxPBoc)), and eventual deprotection (poly­(MeOxP_H2_)^+^). (a) The first-order kinetics graphical interpretation; (b) TGA and DTA analysis of poly­(MeOxPBoc); (c) DSC graphs determining the glass-transition temperatures (*T*_*g*_). Reproduced with permission, Copyright 2022, American Chemical Society.^106^

Damonte et al.^107^ performed a bulk alcoholysis, fundamentally similar to the previously described transesterification promoted by methanol/ethanol. However, the branched PHA oligomers were produced in a subsequent study. The authors modified a particular PHA copolymer, poly­(3-hydroxybutyrate-*co*-3-hydroxyhexanoate) (PHBH), by depolymerizing it with pentaerythritol (PE) catalyzed by zinc stearate (Zn­(St)_2_) at 195 °C for 60 min (displayed in Figure 4). Numerous methods were used to characterize the PHBH branched oligomers produced, including FT-IR, ^1^H NMR, GPC, DSC, and TGA. The PHBH oligomer properties varied with the differing PE content (the authors investigated 2, 5, and 10 wt % of PE). While the thermal stability, as measured by TGA, remained practically unchanged, DSC measurements revealed a decrease in the glass transition temperature with increasing PE content (from −2 °C for pure PHBH to −16 °C for PHBH depolymerized with 10 wt % of PE). The measured chain lengths (by GPC) uncovered the continuous oligomer molecular weight decrease with the total PE content (2 wt % of PE – *M*_n_ = 2270 g/mol, *M*_w_ = 4690 g/mol, D̵ = 2.06; 5 wt % of PE – *M*_n_ = 1790 g/mol, *M*_w_ = 3120 g/mol, D̵ = 1.74; 10 wt % of PE – *M*_n_ = 1350 g/mol, *M*_w_ = 1960 g/mol, D̵ = 1.44). Both FT-IR and ^1^H NMR verified the incorporation of PE into PHBH’s chemical structure. After the depolymerization, the authors prepared PHBH-based porous films from the synthesized branched oligomers (20 wt %) and pure PHBH (80 wt %). Cyrene (dihydrolevoglucosenone), exemplifying a biobased solvent, was used to dissolve the polymer mixture. Several analyses were conducted to characterize the porous films produced, including gravimetric porosity, SEM, dye retention tests, water uptake, tensile tests, and enzymatic hydrolysis. As the authors conclude, the prepared films exhibited decreased elongation at break (dropped from 19.2 ± 2.9% for pure PHBH film to 6.3 ± 0.1%); however, the thin films were efficient cationic dye absorbers, successful Pd^2+^ ion carriers, and exhibited better enzymatic degradability, which could potentially increase their sustainable character.

**Figure 4:**
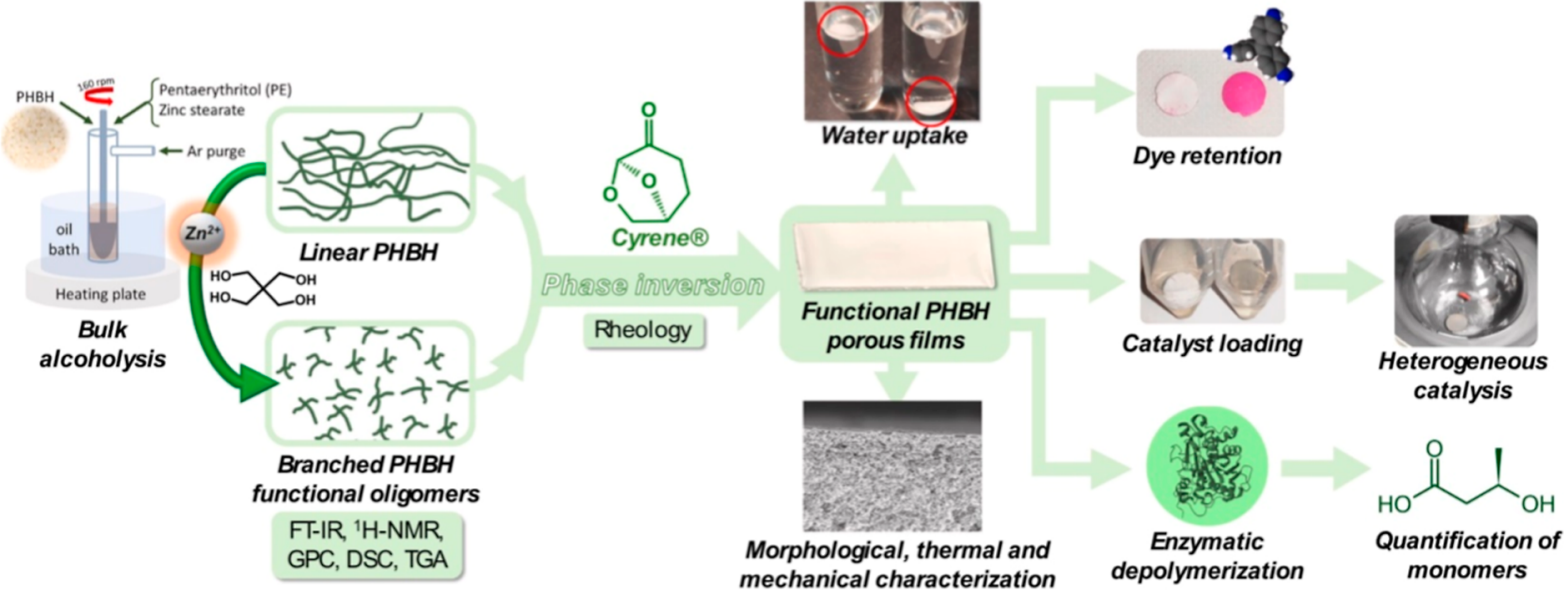
PHA upcycling approach using poly­(3-hydroxybutyrate-*co*-3-hydroxyhexanoate) (PHBH) and pentaerythritol (PE) to prepare branched PHA-derived oligomers suitable for the fabrication of porous functional films with boosted enzymatic degradability and cation retention. Reprinted or adapted with permission under a Creative Commons CC-BY from ref 107. Copyright 2025 Elsevier.^107^

Regarding PHA-upcycled polymeric systems, the eventual mechanical performance is essential for potential upscaling and final utility. Some PHA representatives, specifically poly­(3-hydroxybutyrate), exhibit high crystallinity and a low glass transition temperature (around 5 °C).^182^ Their eventual properties in the produced upcycled systems may exhibit undesired variability at ambient temperatures, which limits their application. The glass transition temperature between 5 and 35 °C poses serious issues, as the material’s properties vary significantly.^183^ The previously reported PHA upcycling routes belong to such materials; therefore, potential valorization strategies that improve the mechanical profile, either to highly flexible elastomers or to rigid thermosets with increased rigidity, would enhance the applicability of engineered compounds based on waste and spoiled PHAs. From a molecular engineering standpoint, higher cross-linking functionality, the presence of polar functional groups (amine, hydroxyl, urethane), or specific aromatic carbon backbones would alter the final material character. The summarized upcycled PHA products are displayed in Table 1.

**Table 1:** PHA Upcycling Approaches Produce Different Added-Value Compounds

produced compound	chemical approach	reacting agent	catalyst	yield	reference
bicyclic ether-ester monomers	alcoholysis, hydrolysis, and nucleophilic substitutions	ethanol, cyclohexane oxide, water	H_2_SO_4_, BF_3_·Et_2_O, NaOH, DMAP, Sn(Oct)_2_, BnOH, TsOH	final conversion 38%	^105^
poly(amine-*alt*-ester)	thermal depolymerization, addition, nucleophilic substitution, cyclization, ROP	ethanolamine, *t*-butyloxycarbonyl compounds	MgO, Et_3_N, DMAP, Sn(Oct)_2_, ZnCl_2_, BnOH	final conversion 43%	^106^
PHA oligomers	alcoholysis	pentaerythritol	Zn(St)_2_	quantitative	^107^

## 3 Upcycling of Polylactic Acid (PLA)

PLA, as a homopolymer polyester, comprises monomer 2-hydroxypropanoic acid linked via intermolecular bonding. Similar to PHAs, particularly to P3HB, the chemical stability of PLA is determined by its ester character.^108^ Although polylactic acid exhibits considerably higher chemical stability than PHAs,^109^ manifested by PLA’s complicated spontaneous composting at mild conditions (applying specific conditions, such as higher heat (at least 50–60 °C) and higher humidity levels (starting at 60%), is mandatory to compost PLA sufficiently),^110^ together with better thermal stability compared to PHAs (the main thermal degradation point reaches around 300 °C for PLA compared to P3HB’s thermal degradation limit of around 180–190 °C),^23,87,111^ even PLA undergoes several degradation mechanisms during its application.^112^ Hydrolysis is a critical process affecting PLA’s structure and overall utility.^113,114^ Since most polylactic acid polymers exhibit significant amorphous character (especially racemic polymers containing both stereoisomers), the hydrolytic processes are more under conditions above the glass transition temperature (around 55–60 °C) due to the polymers’ much lower rigidity.^115^ Many other parameters critically affect PLA’s long-term stability, including the pH levels (typically the acidic conditions accelerate the ongoing hydrolysis together with the autocatalytic boost provided by the released lactic acid),^116^ the polymer’s molecular weight (the higher the molecular weight, the slower the degradation process),^117^ and also the presence of specific enzymes.^118^ The enzymatic presence increases the degradation processes tremendously, especially particular microbial esterases.^118,119^ Since PLA finds utility across many material fields due to its biocompatibility, such as in polymeric scaffolds and medical applications and in environmentally relevant applications, such as packaging and personal use, living organisms and environmental conditions involve the presence of several active proteins.^184,185^ The reported literature emphasizes proteases, lipases, cutinases, and esterases. Such enzymes significantly affect the depolymerization of PLA.^118^ The cold crystallization is another adverse property of initially amorphous PLA.^120^ Similarly to polyethylene terephthalate (PET), polylactic acid suffers from secondary crystallization that is driven thermodynamically^121^ and can occur after the main crystallization when specific conditions are reached (temperature above *T*_*g*_ around 100–130 °C, presence of impurities or additives, and specific processing history—a rapidly cooled polymer structure remains more amorphous).^122^ Due to the properties and characteristics of PLA, upcycling may provide an alternative approach to dealing with a potentially spoiled or waste polymer no longer suitable for its primary purpose.

Upcycling PLA to produce value-added monomer low-molecular-weight compounds is one of the most investigated, studied, and reported processes, as evidenced by multiple literature articles. The conversion to alanine has been reported numerous times by using several different approaches. Photocatalysis is often used to convert the polyester structure into a modifiable lactic acid monomer, which can be transformed into the amino acid or to directly aminolyze the initial polymer. Zhang et al.^123^ used two different catalytic systems based on sulfur/cadmium vacancy-driven C–N coupling, which outperformed the vacancy-free cadmium sulfide (CdS) (see Figure 5). The authors concluded that sulfur vacancies were less effective than cadmium in activating lactic acid but more effective at weakening the adsorption of specific observable radicals (C_α_^•^ and NH_2_^•^). The alanine formation rate promoted by the sulfur-vacancy-containing CdS system achieved 72.5 mmol·g^–1^·h^–1^ for continuous synthesis during 72 h. Another investigation used precisely the same catalytic system for the synthesis of alanine from waste PLA (see Figure 5).^124^ This approach involves ammonolysis and hydrolysis of PLA, resulting in ammonium lactate, which was subsequently converted to alanine over a CdS system with sulfur vacancies. The results confirmed the previously observed behavior: sulfur vacancies provided the highest photocatalytic performance. Unlike previous approaches, Ma et al.^126^ converted PLA to alanine via a multistep chemical route under mild conditions (see Figure 5). Initially, the authors converted the waste PLA packaging and cutlery into lactate form via complete depolymerization in an aqueous potassium hydroxide solution at 60 °C. Then, the pulsed electrooxidation followed, producing pyruvate from lactate (yielding 81.5%), and eventually, alanine was synthesized from pyruvate and hydroxylamine through galvanostatic electroreduction (yielding 84.7%). The overall alanine yield achieved 69.0%, provided on a multigram scale.

**Figure 5:**
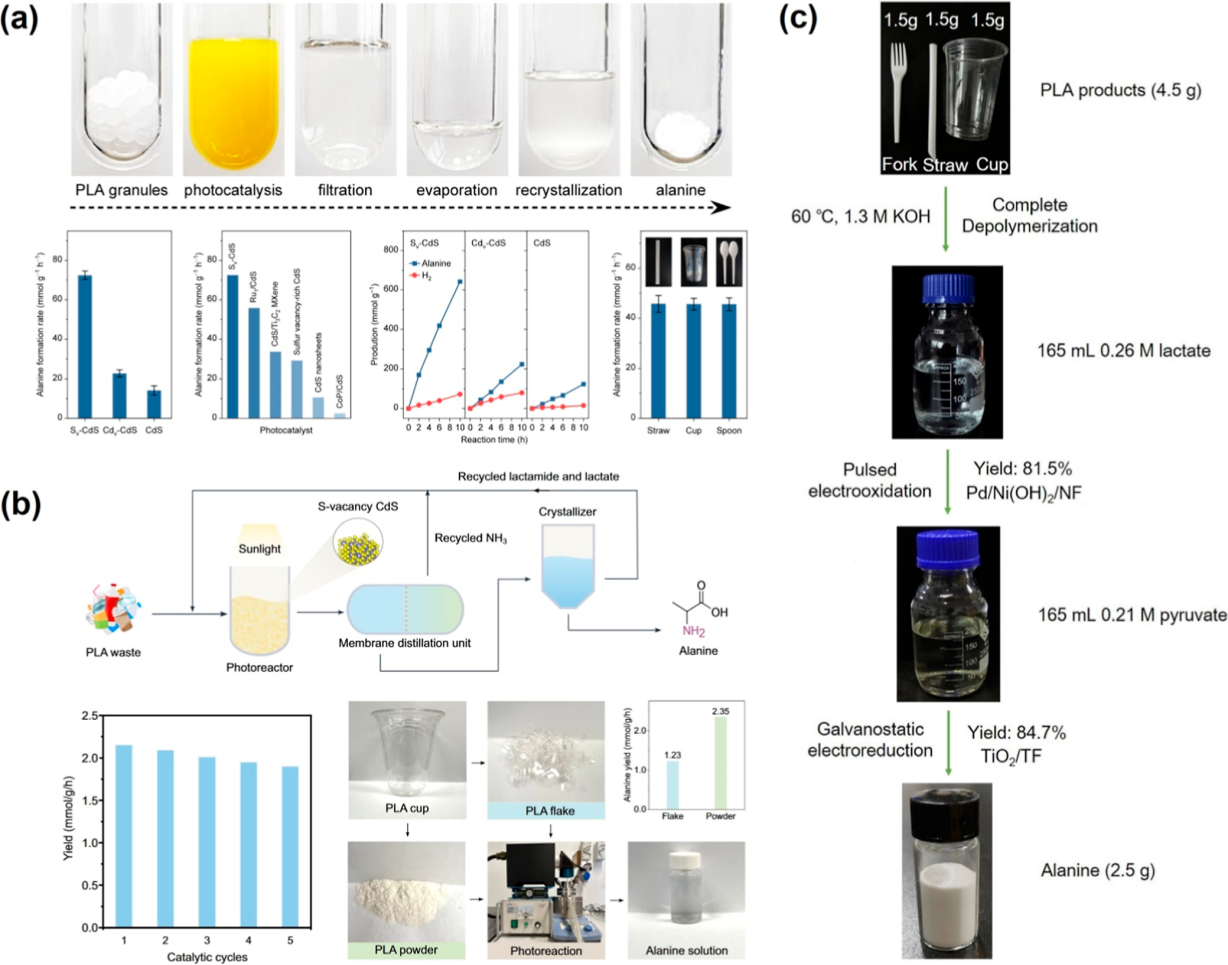
(a) Photocatalytic conversion of waste PLA into alanine through sulfur and cadmium vacancy-driven modification. Reproduced with permission, Copyright 2025, American Chemical Society.^123^ (b) The multicycle valorization of PLA waste producing alanine by photocatalytic sulfur-vacancy cadmium sulfide (CdS). Reprinted or adapted with permission under a Creative Commons CC-BY-NC-ND 4.0 from ref 124. Copyright 2025 Nature Portfolio.^124^ (c) The multistep chemical upcycling of waste PLA packaging and cutlery to alanine through the complete alkaline depolymerization, electrooxidation, and eventual electroreduction. Reproduced with permission, Copyright 2024, The Royal Society of Chemistry.^126^

Next to alanine as an added-value amino acid, other investigations focused on the conversion of PLA into pyruvate/pyruvic acid as an alternative low-molecular-weight upcycled product. Pyruvic acid has potential applications in several fields including pharmaceuticals, cosmetics, and biotechnology. Zhang et al.^129^ reported a photocatalytic upcycling approach that produced pyruvic acid with molecular hydrogen (H_2_) provided to cobalt phosphide (CoP) nanoparticles anchored by cadmium sulfide (CdS) nanosheets. The authors achieved an H_2_ evolution rate of 270 μmol g_cat_^–1^·h^–1^, and the formed pyruvic acid yielded 91.7%. Another study investigated PLA upcycling via a one-pot photothermal approach, producing pyruvic acid and H_2_.^130^ Their process involved waste PLA processed with several compounds with catalytic activity—Pt/A-V-PCN (atomic-layered g-C_3_N_4_), Pt/TiO_2_, and Pt/CdS. The authors successfully performed the PLA conversion, achieving 98.1% selectivity toward pyruvic acid, and subsequently, they reached a H_2_ production rate of 200 ± 17.5 mmol·g_cat_^–1^·g_plastic_^–1^.

Both introduced alanine and pyruvic acid require redox changes to the monomeric lactic acid. Pyruvic acid is generated by selective oxidation of the hydroxyl group occurring on the C2 carbon, while alanine contains an amino functional group instead of the hydroxyl group, typically undergoing reductive amination. These processes require several photo/thermocatalytic systems to achieve such molecular conversions. Many of these processes yield nonquantitative results, unlike, for example, complete alkali depolymerization. Since lactic acid is the PLA monomer, appropriate alcoholysis yields numerous lactate monoesters, which are applicable in cosmetics or as industrial solvents and cleaning agents. Xie et al.^128^ demonstrated chemical upcycling of PLA plastic waste to methyl lactate, catalyzed by specific ionic liquid (tetramethylammonium fluoride (TMAF)) (see Figure 6). TMAF was selected since it achieved the best product yield (close to 60%) compared to other quaternary ammonium salts (ethyl fluoride salt (TEAF) reached 44% yield; methyl chloride salt (TMACl) resulted in around 3% yield). The authors also performed five catalyst reuse runs, confirming the maintenance of catalytic activity. It was observed that the ammonium salts containing fluoride anions dissociated in methanol (the depolymerization alcohol), as confirmed by Raman spectroscopy, suggesting potentially significant catalytic activity, according to the authors. They also conclude that the complete methanolysis occurred after 60 min of the reaction time at 90–110 °C. Hubble et al.^135^ also performed a successful PLA upcycling using a custom ionic liquid catalyst synthesized from N,N-dimethylglycine and 1,5,7-triazabicyclo[4.4.0]­dec-5-ene (see Figure 6). The authors achieved >93% conversion of PLA to methyl lactate after 3 h at 70 °C. Unlike the previous study, the ionic liquid catalyst contained nitrogen atoms rather than fluoride anions; therefore, alcoholysis depolymerization occurred under several reaction conditions.

**Figure 6:**
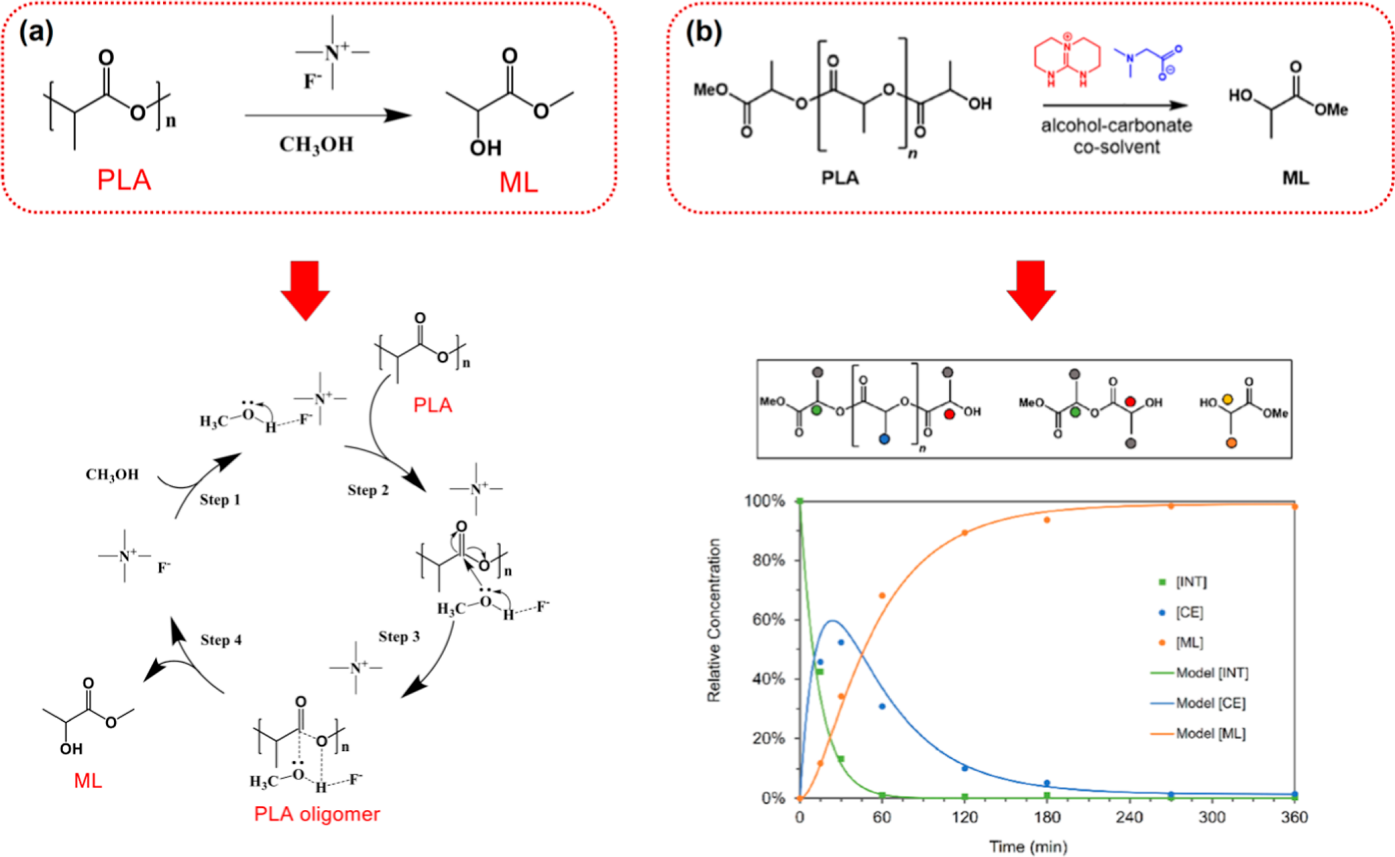
PLA upcycling approaches producing lactate esters catalyzed by selected ionic liquids. (a) The methanolysis of waste PLA catalyzed by tetramethylammonium fluoride (TMAF). Reproduced with permission. Copyright 2021, Elsevier.^128^ (b) The methanolysis of waste PLA catalyzed by the synthesized catalyst from (N,N-dimethylglycine) and (1,5,7-triazabicyclo[4.4.0]­dec-5-ene). Reprinted or adapted with permission under a Creative Commons CC-BY 4.0 from ref 135. Copyright 2023, American Chemical Society.^135^

In addition to PLA conversion to alanine and pyruvic acid, many other monomer products and derivatives were suggested and produced. Makarov and Rueping^131^ studied the alcoholysis and aminolysis of PLA, producing numerous alkyl lactates and lactamides. Such products find their purpose in pharmaceutics and cosmetics and represent an environmentally safer solvent for agriculture and bioprocesses. The study investigated three sources of depolymerized polylactic acid: virgin polymer, waste PLA cups, and scrap PLA 3D-printing filaments. Next to the standard reaction conditions (the authors report 35–40 °C), resonant acoustic mixing was applied during the depolymerization process. In total, five different alcohols and amines (ten eventual products) were synthesized, achieving 79% yield at the lowest (aminolysis with isopropylamine) and 94% yield at the highest (methyl lactate). Not only can the upcycling approach produce various low-molecular-weight products, but choosing the appropriate depolymerization strategy also ensures selective product isolation. Spicer et al.^132^ demonstrated sequential catalytic chemical depolymerization involving polylactic acid, bisphenol A polycarbonate (BPA-PC), and polyethylene terephthalate (PET). Different reaction conditions (120, 150, and 180 °C) and seven catalytic systems (metal chlorides and acetates) were used to achieve polymer upcycling. The cascade depolymerization and separation process involved three steps. Initially, the PLA–BPA-PC–PET mixture was homogenized with a selective PLA-reacting catalyst (magnesium chloride) and ethylene glycol, producing 2-hydroxyethyl lactate selectively. The PLA-depolymerization product was separated, leaving a BPA-PC–PET mixture. Then, imidazole was added with an additional amount of ethylene glycol to depolymerize bisphenol A polycarbonate, yielding two products: bisphenol A and ethylene carbonate. Lastly, zinc acetate was mixed with the leftover PET with additional ethylene glycol, producing bis­(2-hydroxyethyl) terephthalate (BHET) as the final upcycling product.

Several other low-molecular-weight molecules were suggested, investigated, and characterized in published studies. In addition to the compounds mentioned earlier, the synthesized value-added molecules, together with the chosen strategy, reaction conditions, yields, and references, are summarized in Table 2.

**Table 2:** PLA Upcycling Approaches Produce Different Added-Value Compounds

produced compound	chemical approach	reacting agent	catalyst	yield	reference
alanine	photocatalytic C–N coupling	H_2_O, ammonia	S- and Cd-vacant CdS	72.5 mmol ·g^–1^·h^–1^	^123^
alanine	photocatalytic conversion	H_2_O, ammonia	S-vacant CdS	5 mmol ·g^–1^·h^–1^	^124^
alanine	photocatalytic conversion	H_2_O, ammonia	Ni/ZnIn_2_S_4_	61.91 mmol ·g^–1^·h^–1^	^125^
alanine	pulsed electrooxidation, galvanostatic electroreduction	H_2_O, hydroxylamine	KOH, Pd/Ni(OH)_2_, TiO_2_	69%	^126^
alanine	catalytic amination	H_2_O, ammonia	Ru/Ce_3_In-MMO	76.1%	^127^
alkyl lactate	alcoholysis	methanol	tetramethylammonium fluoride (TMAF)	59%	^128^
alkyl lactates, lactamides	alcoholysis, aminolysis	methanol, isopropanol, butanol, isobutanol, 3-buten-1-ol, butylamine, isopropylamine, isobutylamine, cyclopropylamine, allylamine	triazabicyclodecene (TBD)	79–94%	^131^
alkyl lactate	glycolysis	ethylene glycol	MgCl_2_	97–99%	^132^
acetic acid	photocatalysis	water	(FeCoNiCuZn)WO4, denoted XWO4	38.51 mg·g^–1^·h^–1^	^133^
lactide	photocatalytic hydrolysis	water	α-Fe_2_O_3_	32%	^134^
pyruvic acid	photocatalysis	water	CoP nanoparticle-anchored CdS	91.7%	^129^
pyruvic acid	photo/thermal reaction	water	Pt/TiO2, Pt/A-V-PCN, and Pt/CdS	274.13 mg·g_PLA_^–1^	^130^

Previously discussed strategies aimed to convert PLA waste into chemically modified particular low-molecular-weight products applicable in several industry fields. Also, all of the processes involved the complete depolymerization of PLA via several chemical mechanisms, such as hydrolysis, alcoholysis, or aminolysis, followed by particular functionalization. The polylactic acid polymeric waste can also be valorized into products with specific application potential in solid-state form, avoiding chemical structural changes. Feroce et al.^136^ turned postconsumer PLA scrap into active packaging films by incorporating polycaprolactone (PCL) into polylactic acid. Taking the polymeric structure of PLA into account during the upcycling process yields several advantages. The complete depolymerization increases the valorization cost and requires several chemical and hardware instruments. Additionally, incorporating PLA into a polymer solid state without chemical modification avoids the structural changes that can potentially form volatile, hazardous, or nonselective products and byproducts. The published work investigated combining PLA and PCL to develop new active packaging systems from secondary waste sources. The authors prepared polymeric films comprising different PLA/PCL blends (85/15 and 70/30 wt %), which were enhanced with quercetin (Q) (3 wt %), exemplifying a natural polyphenolic antioxidant that stabilizes the prepared polymer system. The authors reported that the prepared blends exhibited much higher crystallinity (∼25% for the 70/30 wt % system) compared to the virgin PLA (5.3%). Also, the PLA/PCL 70/30 wt % achieved the best overall performance and the most potent antioxidant effect (provided by quercetin). Liu et al.^137^ synthesized a magnesium-based metal–organic framework (Mg-MOF) via a multistep approach combining mechanochemical milling and solution mixing, which was subsequently transformed into crumpled carbon nanosheets (CCNs). The achieved CCN possessed a thickness of 2.5 nm and abundant nanopores and oxygen-containing groups. The target application of the prepared upcycled PLA-derived CCNs is functional materials for potential energy conversion and storage or environmental remediation.

The most complex strategies involve multistep transformations of PLA into specific reactive intermediates used to fabricate new materials. The unusable PLA, with a low molecular weight or nonideal properties for the original application, can be specifically modified into oligomer structures with vacant hydroxyl groups suitable for the chemical modification (see Figure 7). Shao et al.^138^ demonstrated a complex PLA upcycling strategy leading to 3D printing materials. The authors valorized FDM 3D printing PLA scrap via catalystless aminolysis with ethanolamine (EA) as the nucleophile (five different PLA/EA ratios were investigated at varying molar ratios). According to the conclusion, the PLA/EA ratio of 1:4 at 100 °C after 60 min of aminolysis achieved 100% degradation degree. The formed N-lactoyl ethanolamine (N-LEA) exemplified a reactive intermediate containing two vacant hydroxyl groups suitable for further modification. Eventually, N-LEA was modified by methacrylic anhydride, forming the N-LEA dimethacrylate ester (DME). Due to the presence of the amide functional group, DME formed a solid-phase powder; therefore, an appropriate reactive diluent, 4-acryloylmorpholine (ACMO), served as a solution-forming additive. The prepared DME/ACMO systems (with varying reactive diluent contents) were 3D-printed and characterized using several methods (FT-IR, DSC, tensile testing, impact strength, and TGA). According to the results, the prepared DME/ACMO 3D-printed resins outperformed the commercially available resins (produced by Anycubic and Monoprice). Another amide-forming strategy was investigated by Liu et al.,^139^ leading to hydroxypropanamide derivatives. The authors also synthesized methyl lactate during the upcycling process. The final PLA monomer products were repolymerized into polyamide polymers (PEA) with considerable molecular weights (up to 29,800 g/mol) and glass transition temperatures ranging from 12.8 to 117.6 °C, demonstrating impressive thermal stability (due to amide’s thermodynamically stable structure).^140^

**Figure 7:**
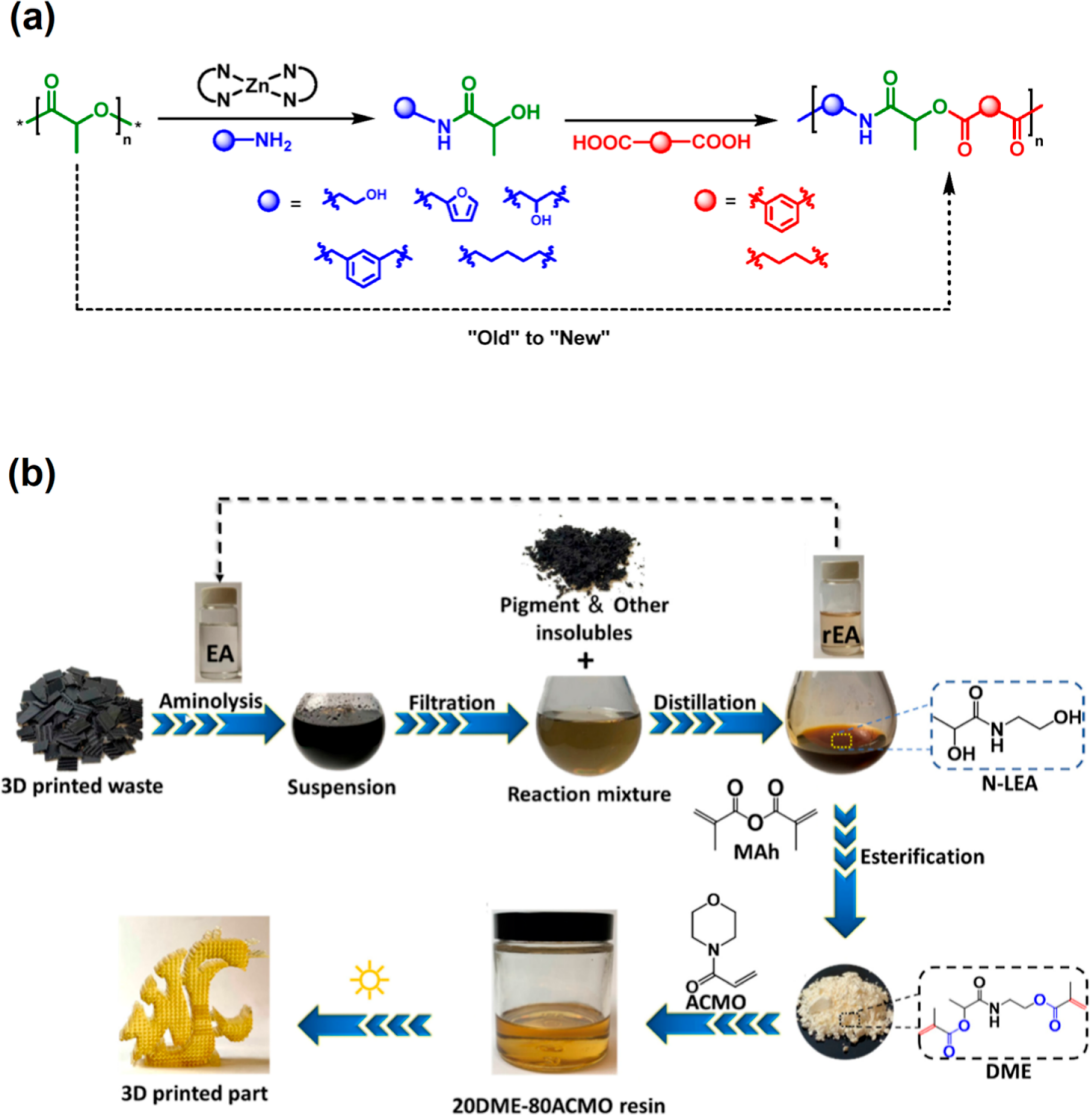
Two different PLA upcycling strategies yield lactate–amide reaction intermediates for further modification. (a) Depolymerization of PLA over a zinc catalyst produces several lactamides used for repolymerization to polylactamides. Reproduced with permission. Copyright 2024, American Chemical Society.^139^ (b) Chemical upcycling approach converting FDM 3D printing waste PLA into N-lactoyl ethanolamine (N-LEA), further modified by methacrylic anhydride (MAh) to N-LEA dimethacrylate ester (DME) suitable for reactive precursor fabrication applicable in SLA 3D printing. Reproduced with permission. Copyright 2022, The Royal Society of Chemistry.^138^

## 4 Upcycling of Polybutylene Succinate (PBS)

Polybutylene succinate (PBS) is a polymer made from 1,4-butanediol and succinic acid and can be produced from renewable sources via biotechnological processes.^141^ In 2021, a total of 87,000 tons of biobased PBS were produced, representing 3.5% of total biobased polymer production.^142^ The biodegradability and the flexible mechanical profile contribute significantly to the packaging sector and agriculture.^51^ The enzymatic hydrolysis exemplifies the primary degradation mechanism of PBS performed by specific microorganisms, such as bacteria (*Bacillus*, *Acinetobacter*, *Pseudomonas*, and *Terribacillus* species)^143–145^ or fungi (*Aspergillus*, *Penicillium*, and *Fusarium*),^146,147^ and lipases or cutinases are the primary enzymes responsible for the hydrolysis.^148,149^ Next to the limited thermal stability associated with PBS’s biodegradable chemical structure, this polyester exhibits relatively low melt strength, which complicates the plastics processing from a rheological standpoint.^150,151^ Commonly, PBS can impart its flexibility to other polyester polymers, such as PLA or PHAs.^77,152^ The direct chemical upcycling and valorization of degraded and waste PBS represents a unique option for decreasing the potential environmental pollution linked to the excessive presence of PBS. Although polybutylene succinate degrades in the environment, its incorporation into value-added products increases its applicability. Moreover, several upcycling depolymerizations, such as alcoholysis, hydrolysis, and aminolysis, selectively react with PBS in heterogeneous systems containing numerous impurities and adulterants.^161,162,166^ The formed monomeric products or isolable structures are purified from the nonreactive components and serve particular new purposes.^153,154^ This approach is much less constrained by heterogeneous components than mechanical recycling. Not only is PBS thermally unstable, but the potential polymer used in agricultural applications would also contain adulterants, such as dirt, sand, and other soil components.^155,156^ The upcycling of PBS represents a prospective strategy leading to polyester valorization, avoiding the primary biodegradation in the environment, which should exemplify the last option toward sustainability.

Several published studies have focused on the biobased synthesis of PBS or on the closed-loop depolymerization of this polyester to produce monomer precursors. The closed-loop PBS polymerization, hydrolysis, and repolymerization process was demonstrated by Papaspyrides et al.^157^ The authors synthesized a PBS prepolymer, which was subjected to a solid-state polymerization (SSP) for 29 h under a nitrogen atmosphere. Primarily, the melting point was observed and tracked during polymer synthesis, starting at 112–114 °C and rising to 128 °C. The melting point of the synthesized polymer generally indicated an increase in molecular weight of the growing carbon chain.^158^ The authors eventually state that SSP is a promising PBS recycling technique, as it “revives” the hydrolyzed polybutylene succinate structure. Apart from the closed-loop PBS production, which is rather chemical recycling than upcycling, as demonstrated also by different research groups,^159,160^ Xiong et al.^161^ investigated a sustainable and economically viable approach to converting PBS waste plastic into a monomeric succinic acid (SA) with exceptional purity. The researchers applied a complex chemical strategy involving paired electrocatalysis, using a thiol-engineered metal–organic framework (NiBDC@NiS_0.68_) catalyst coupled with CO_2_-assisted precipitation. This approach is an alternative to the current electrocatalytic conversion pathway, which has critical limitations, such as a costly purification process and low Faradaic efficiencies. The proposed metal–organic frameworks, including the process together with carbon dioxide precipitation, achieved an SA yield of 92.5%, exhibiting high purity and coproducing sodium hydrogen carbonate. While SA can be applied either for continuous PBS synthesis or in other applications, such as pharmaceutics, cosmetics, or industrial chemistry, the upcycling of PBS described by Wu et al.^162^ demonstrates the production of succinimides from waste polybutylene succinate over succinimide anion-based ionic liquids, such as 1,8-diazabicyclo[5.4.0]­undec-7-ene succinimide, [HDBU]­[Suc]. The synthesized succinimides find application in polymer science to enhance thermal stability and mechanical performance, as nitrogen-containing compounds form stronger intermolecular noncovalent bonding, thereby improving physical–chemical properties.^163^ Succinimides also play a significant role in organic synthesis, analytical chemistry, cosmetics, and pharmaceuticals.^164,165^ The authors concluded that the optimal reaction temperature is around 130 °C, and a negligible amount of water in the reaction mixture is beneficial due to the better function of the [HDBU]­[Suc] catalyst. Several particular succinimide products were synthesized as illustrated in Figure 8.

**Figure 8:**
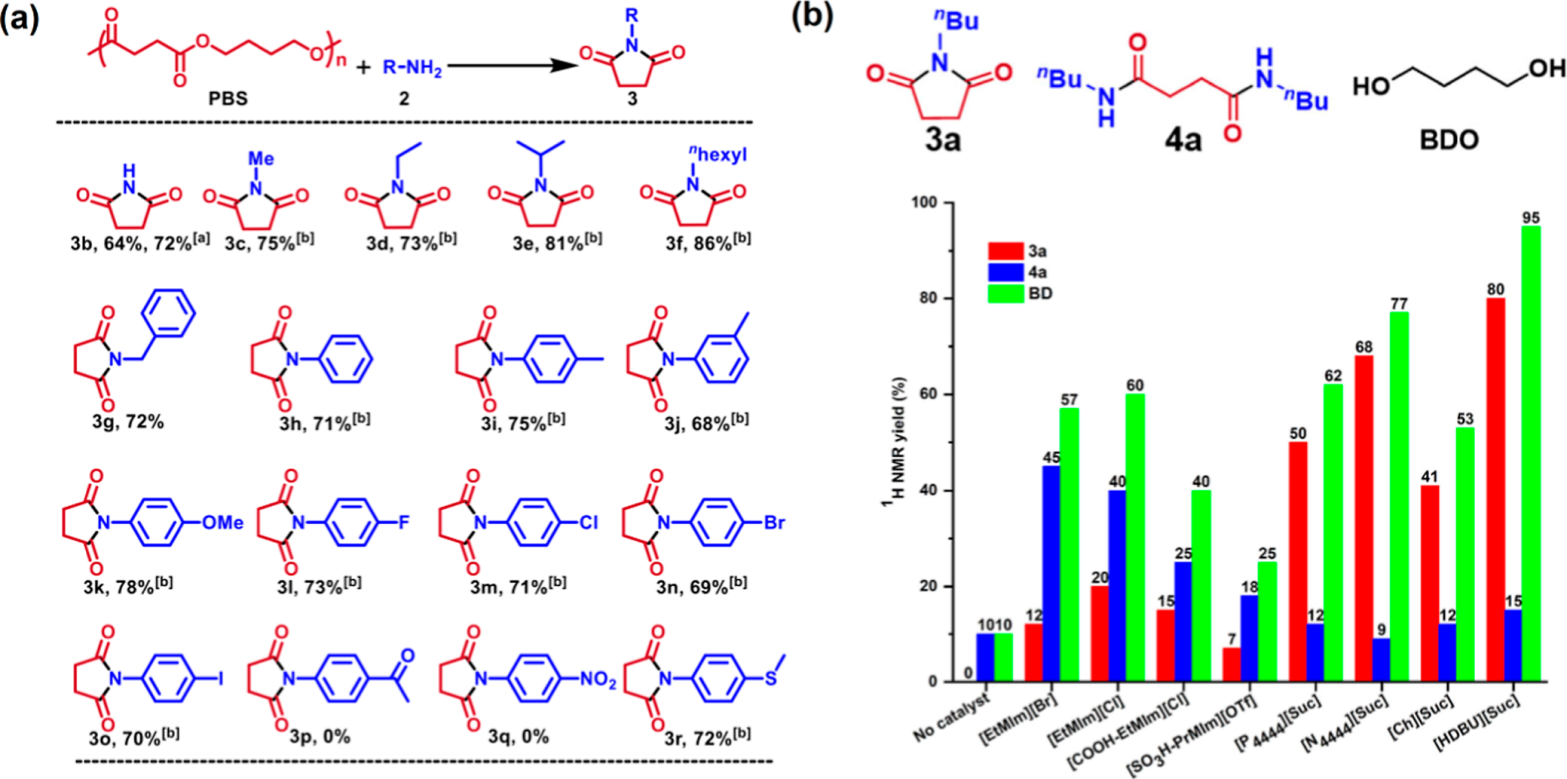
Polybutylene succinate upcycling approach producing succinimide compounds. (a) The reaction scheme describes the chemical pathway to the product and the upcycled compounds along with the corresponding yields. (b) The efficiencies of the investigated ionic liquid catalysts mark the best performance of 1,8-diazabicyclo[5.4.0]­undec-7-ene succinimide, [HDBU]­[Suc]. Reprinted or adapted with permission under a Creative Commons CC-BY 4.0 from ref 162. Copyright 2024, Nature Portfolio.^162^

Lourenço et al.^167^ demonstrated an interesting strategy for upcycling polybutylene succinate (see Figure 9a,b). The authors showed specific depolymerization pathways for PBS and P4HB to 1,4-butanediol, which serves as an intermediate in the synthesis of busulfan. Several catalytic systems were applied to produce the eventual upcycled compounds, namely, MoO_2_Cl_2_(H_2_O)_2_/TMDS, MoO_2_Cl_2_(H_2_O)_2_/HBpin, and KOH/PhSiH_3_. Busulfan, 4-methylsulfonyloxybutyl methanesulfonate, has been an anticancer drug used in pharmacies since 1959.^167–169^ The authors demonstrated the synthesis of 1,4-butanediol followed by the preparation of busulfan, achieving 54% and 51% yields from P4HB and PBS, respectively. The study investigated the optimal catalytic system for the proposed upcycling strategy. A physically aged polybutylene succinate was converted into PBS-based vitrimers via another chemical approach (Figure 9c).^172^ The authors prepared two sets of semicrystalline PBS-based vitrimers, cross-linked with diglycidyl ether of bisphenol A (DGEBA) or glycerol (a sustainable cross-linker) in the presence of a Zn­(II) catalyst. Vitrimers are a specific type of polymer that exhibit a strong, thermoset molecular character at low temperatures but can be reshaped and recycled at high temperatures due to their dynamic covalent bonding structure.^170,171^ The authors investigated different cross-linker loadings (1–5 and 10 mol %) processed at different reaction temperatures (150, 160, and 170 °C). The produced vitrimers exhibited high crystallinity (45–60%), moderate melting temperatures (109.7–114.5 °C), high insolubility (50–92%), and enhanced melt strength (a four-order-of-magnitude increase over the conventional PBS). Although DGEBA contains two primary reactive oxirane functional groups, the secondary-formed hydroxyl reacts with carboxyls/hydroxyls, resulting in a higher overall cross-linking density over the glycerol cross-linker. We summarized all the discussed upcycling approaches valorizing PBS in Table 3.

**Figure 9:**
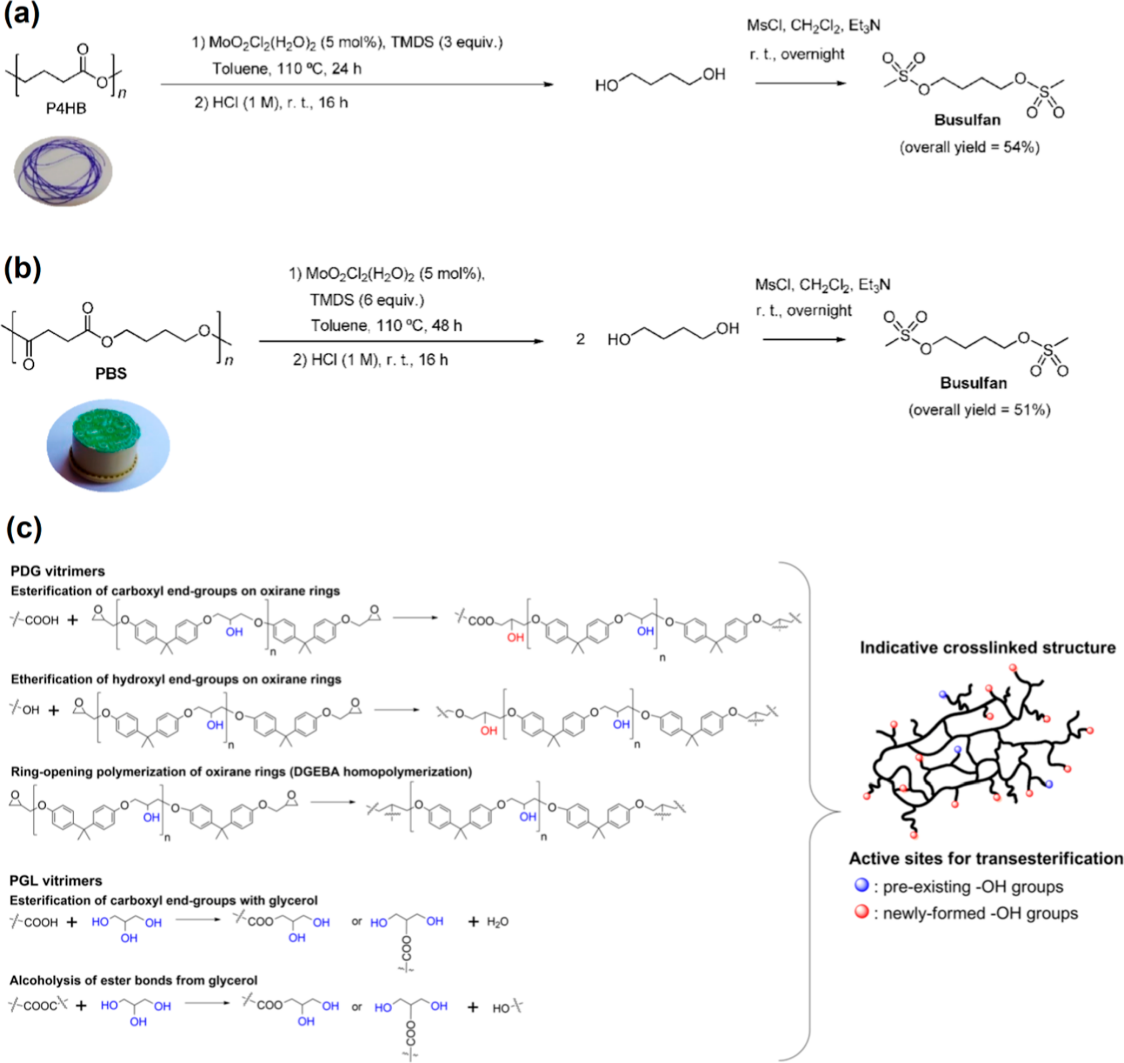
(a) The poly­(4-hydroxybutyrate) upcycling to busulfan (4-methylsulfonyloxybutyl methanesulfonate). Reprinted or adapted with permission under a Creative Commons CC-BY 4.0 from ref 167. Copyright 2022, MDPI.^167^ (b) The poly­(butylene succinate) upcycling to busulfan.^167^ (c) PBS-based vitrimers synthesized from the physically aged polybutylene succinate, using diglycidyl ether of bisphenol A (DGEBA) and glycerol as a cross-linker dynamically incorporated into the material by a Zn­(II) catalyst. Reprinted or adapted with permission under a Creative Commons CC-BY 4.0 from ref 172. Copyright 2024, American Chemical Society.^172^

**Table 3:** PBS Upcycling Approaches Produce Different Added-Value Compounds

produced compound	chemical approach	reacting agent	catalyst	yield	reference
PBS monomers	alkali hydrolysis	water	NaOH, Na_2_CO_3_	depolymerization 97.3%	^159^
PBS monomers	catalytic hydrolysis	water	succinic acid	depolymerization about 90%	^160^
succinic acid	alkali hydrolysis, electrocatalytic strategy	water, water electrolytes	alkali, NiBDC@NiS0.68 heterojunction catalyst	92.5%	^161^
N-substituted succinimides	aminolysis	amines	anion-based ionic liquids	69–86%	^162^
busulfan	reductive depolymerization	water	MoO_2_Cl_2_(H_2_O)_2_/TMDS, silane, HCl	48–75%	^167^
PBS vitrimers	vitrimerization	glycerol, DGEBA	Zn(II) catalyst	quantitative	^172^

## 5 Conclusion and Future Outlook

This proposed review summarizes the most widely used biobased bioplastics across several industrial segments. Modern society is heading toward a sustainable future, where polymeric materials derived from renewable sources play an essential role as functional substitutes for fossil-based compounds. We focused on three biobased polymer groups: the biosynthesized polyhydroxyalkanoates (PHAs) produced by several microbial producers via in vivo biosynthesis. PHAs are a large group of particular polymers; however, poly­(3-hydroxybutyrate) (P3HB) and poly­(4-hydroxybutyrate) (P4HB) and their copolymers are the most well-known and produced derivatives. Polylactic acid (polylactide) (PLA) exemplifies an ester homopolymer fabricated from the biotechnologically produced lactic acid via multistep chemical synthesis. Compared to PHAs, polylactic acid exhibits significantly greater chemical and thermal stability, which favors its conventional thermoplastic applications. However, the used and hydrolyzed PLA fabrics and plastic waste represent a significant carbon source that could be valorized into other valuable products. Polybutylene succinate (PBS) requires two potential biotechnological products for its production—succinic acid and 1,4-butanediol. Both of these reactants are obtainable via a sustainable production route. Due to PBS’s primary application purpose, specifically in agriculture and flexible packaging, its accumulation in the environment might become an issue even with taking PBS’s biodegradability into account. Sustainable polymers with biodegradation potential should be used to the fullest, and their biodegradability should serve as the “last sustainability insurance”.

The summarized waste valorization strategies provide an innovative alternative approach to reducing the potential accumulation of polyester bioplastics in the environment. Given the application fields involving the discussed polyesters, pragmatic suggestions for a closed-loop, sustainable approach to used, spoiled, or leftover polymers are desired not only to enhance future sustainable expectations but also to reduce the expenses associated with virgin-materials-based manufacturers. Polyhydroxyalkanoates exhibit exceptional biodegradation potential, which complicates their potential for material recycling. Additionally, the thermal stability of PHAs is an issue for polymer reprocessing, negatively affecting conventional processes, such as extrusion, molding, or thermoforming. This review summarizes the investigated strategies using waste PHAs to produce functional monomers via alcoholysis or aminolysis. The resulting reactive compounds were used to synthesize structurally novel poly­(ester ether) or poly­(amide ester) polymers suitable for closed-loop chemical recycling. Another approach converted poly­(3-hydroxybutyrate-*co*-3-hydroxyhexanoate) to multifunctional oligomers involving pentaerythritol, yielding branched polyol structures ideal for the production of porous layers.

Polylactic acid, as a homopolymer polyester, consists of lactic acid units linked by intermolecular bonds. Since PLA comprises a single monomer unit, upcycling strategies can focus on the release or chemical modification of the carboxylic acid into building blocks to produce several added-value products. We reported several investigated strategies that generate numerous functional molecules such as alanine, alkyl lactates, lactamides, acetic acid, lactide, and pyruvic acid. Many of the named compounds find application across industrial fields such as pharmaceutics, cosmetics, chemical intermediates, and industrial solvents. In addition to the particular low-molecular products, we reported highly efficient, multistep processes producing thermodynamically stable polyamides containing functionalized lactic acid and another approach involving the aminolysis of postconsumer PLA waste, producing a reactive monomer, lactamide, which is eventually functionalized with methacrylate derivatives to form a curable resin suitable for SLA 3D printing. Finally, the polybutylene succinate upcycling options were discussed, including the synthesis of monomeric succinimides for organic synthesis or analytical chemistry, PBS-based dynamically cross-linked vitrimers, and the production of an anticancer compound, busulfan.

The future outlook should reflect the pros and cons of the chemical upcycling of polyesters and identify suitable source fields for using waste-entering materials in conjunction with economically rational approaches. The complete depolymerization of polyester structures requires excessive amounts of reacting solvents and agents along with specific catalysts. The further repolymerization or application of the low-molecular-weight functional molecules should provide the greatest added value to cover the expenses associated with their production. The sustainability aspect of waste reduction is essential, but modern industry demands solutions that involve economically rational strategies. Valorized/biobased intermediates and upcycled products can serve as suitable alternatives for the thermoset polymer segment. The thermoset polymer cannot be recycled via conventional mechanical recycling, and its chemical valorization is complicated and expensive. Fossil-based entering substances might be replaced by valorized or biobased compounds, which would reduce waste accumulation and conserve nonrenewable resources. Moreover, the engineered upcycled thermoset precursors might be enhanced by vitrimer structures, which could enable closed-loop recycling of generally nonrecycled petrochemical thermosets. Additionally, several bioplastics are blended to fulfill the performance requirements of their target applications. Upcycling strategies can selectively fractionate the valorized low-molecular-weight compounds or yield engineered materials containing modified heterogeneous precursors that can be applied in mixtures. As listed, upcycling is a promising chemical strategy for addressing specific reactive polymers, but sustainability should be linked to economically rational solutions.
